# Influence of prenatal corticosteroid therapy on neonatal vitality and utility as a labor-inducing agent in Santa Inês ewes

**DOI:** 10.1590/1984-3143-AR2022-0109

**Published:** 2024-03-22

**Authors:** Elisiane Sateles dos Santos, Rodrigo Freitas Bittencourt, Gleice Mendes Xavier, Carmo Emanuel Almeida Biscarde, Isabella de Matos Brandão Carneiro, Mateus Martins Rodrigues dos Santos, Antonio de Lisboa Ribeiro

**Affiliations:** 1 Escola de Medicina Veterinária e Zootecnia, Universidade Federal da Bahia - UFBA, Salvador, BA, Brasil

**Keywords:** corticosteroid, parturient, reproductive biotechnology, sheep

## Abstract

Since the 1970s, maternal corticosteroid therapy has been used successfully to induce labor. This allows for better monitoring of parturients and provision of first aid to neonates, improving neonatal viability, as this treatment induces maturation in a variety of fetal tissues, thereby reducing morbidity and mortality. Although the effects of corticosteroids are well known, few studies have investigated the influence of this therapy in Santa Inês sheep. This study aimed to evaluate the efficacy of dexamethasone at two doses (8 and 16 mg) to induce lambing in Santa Inês ewes at 145 days of gestation and assess its effects on neonatal vitality. For this study, 58 ewes raised in an extensive system were investigated. Pregnancy was confirmed after artificial insemination at a set time or after controlled mounting. Ewes were separated into three groups: an untreated control group (G1: 0 mg) and groups treated with two doses of dexamethasone (G2: 8 mg and G3: 16 mg). In total, 79 lambs were born. Their vitality was assessed based on their Apgar score, weight, temperature, and postnatal behavior. SAS v9.1.3 (SAS Institute, Cary, NC) was used to analyze data, considering a 5% significance level for all analyses. The births in the induced groups occurred 48.4 ± 22.1 h after induction, while the ewes that underwent non-induced labor gave birth 131.96 ± 41.9 h after placebo application (p < 0.05), confirming the efficacy of dexamethasone to induce and synchronize labor. The induced and non-induced neonates had similar Apgar scores, temperatures, weights, and postnatal behavioral parameters (p > 0.05). This study showed that inducing labor in Santa Inês ewes at 145 days of gestation with a full (16 mg) or half dose (8 mg) of dexamethasone is an effective technique and does not compromise neonate vitality.

## Introduction

Since the 1970s, many studies have reported on inducing labor in sheep (Adams and Wagner, 1970). This method aims to synchronize parturitions to streamline workers’ labor so that they may provide assistance to sheep and manage neonates, thereby minimizing maternal and neonatal mortality and enhancing breeding efficiency (Özalp et al., 2017). Among the currently available drugs capable of inducing labor in ewes, dexamethasone (DEX) is most often used and has shown to be efficient and inexpensive as compared to other labor-inducing drugs. The dose most commonly used is 16 mg (Kastelic et al., 1996; Ingoldby and Jackson, 2001).

Labor is induced through exogenous glucocorticoids that mimic the action of cortisol produced by the fetal adrenal cortex, which acts as an initial trigger to a hormonal cascade that, in turn, triggers labor (Zoller et al., 2015; Barreto et al., 2021). Corticosteroids also promote fetal maturation, particularly structural pulmonary maturation, thereby stimulating the production of surfactant phospholipids and activating the maturation of brown adipose tissue (Liggins, 1969; Fowden et al., 1995; Ballard et al., 1997; Zaremba et al., 1997; Bispham et al., 2002).

In 1972, in a study by Liggins and Howie (1972) on the artificial induction of fetal lung maturation, premature lambs of mothers who had received antenatal corticosteroid therapy exhibited better vitality than did the lambs of mothers who had not received corticosteroid therapy. Subsequently, new studies were conducted and demonstrated a reduction in pulmonary changes and better rates of neonatal viability for fetuses of ewes that underwent antenatal glucocorticoid therapy (Feitosa et al., 2017; Regazzi et al., 2022).

Assessing neonatal vitality at birth allows for assessment of the neonate’s vital parameters in its first hours of life and the opportunity to implement preventive or corrective methods to the changes found, and to differentiate between healthy and compromised neonates (Garcia, 2001; Vassalo et al., 2014). In veterinary medicine, a neonate’s clinical condition is determined by several adaptations of the Apgar score, a method created by a medical anesthesiologist for application in human neonates (Garcia, 2001). In a recent study, Barreto et al. (2021) found that neonates with a high Apgar score ingested a greater amount of colostrum and had a higher liver enzyme index, which is associated with greater survival likelihood.

Despite prior knowledge of the action of DEX on fetal maturation, relatively few studies have investigated the interaction between corticosteroid-induced labor and parameters associated with neonatal vitality, particularly in Santa Inês lambs. Thus, this study set out to assess the efficacy of two different DEX doses, the full conventional dose (16 mg) and a half dose (8 mg), for inducing labor in Santa Inês ewes at 145 days of gestation, and to analyze the effect of induction on the lambs’ neonatal vitality.

## Methods

The study was conducted on two experimental farms, with the following geographical coordinates: altitude of 162 m and 234 m, an average annual rainfall of 1,251 mm and 1,079 mm, and a hot, semi-humid and a hot, humid climate, for the first and second farm, respectively.

The study was conducted based on the ethical precepts recommended by the National Council for the Control of Animal Experimentation (CONCEA), following approval by the Ethics Committee on the Use of Animals (CEUA) under protocol number 23/2018.

In this study, 58 multiparous adult ewes, with an average weight of 43.34 ± 8.49 were observed and average body condition of 3±0,35, based on a scale of 0 to 5 points (from very thin to very fat) devised by Jefferies (1961). The animals were initially subjected to clinical, gynecological, and ultrasound examinations with the aid of a multifrequency transrectal linear transducer, used at a frequency of 7.5 MHz (Mindray Z5, Shenzhen, China). Clinically healthy ewes that did not present abnormalities in the reproductive tract and were not pregnant at the time of the examination were considered eligible for inclusion in the study.

The ewes were raised in an extensive farming system on pasture (mixed pasture with *Brachiara humidícula* and *B. decumbens*), with water and mineral salt ad libitum, without added concentrates. Before the experiment began, all animals were vaccinated and dewormed.

After the ultrasound examination, the animals were included in an estrus synchronization protocol in October 2016 at both experimental farms. Initially, all animals received an intravaginal device with 0.33 g of progesterone (CIDR®, Pfizer, Guarulhos, Brazil), on a random day of the estrous cycle (Day 0), which remained in place for 8 consecutive days. On day to the removal of the devices, 300 IU of equine chorionic gonadotropin (eCG, Novormon®, MSD Animal Health, São Paulo, Brazil) and 0.125 mg of cloprostenol sodium (PGF2α, Ciosin ®, MSD Animal Health) were administered via intramuscular (IM) injection. Thirty-two hours after CIDR removal, 200 IU of human chorionic gonadotropin (hCG, Chorulon ®, MSD Animal Health, São Paulo, Brazil) was applied (Kiya et al., 2017). Considering that the average interval between CIDR removal and the end of estrus is 60 h and knowing that ovulation in sheep preferentially occurs at the end of estrus (Gordon, 1997), laparoscopic intrauterine insemination was performed with frozen semen from a reproduction center approximately 56 h after removal of the intravaginal device. The semen parameters exceeded those recommended by the Brazilian College of Animal Reproduction (CBRA) (Henry and Neves, 2013).

**Figure 1:**
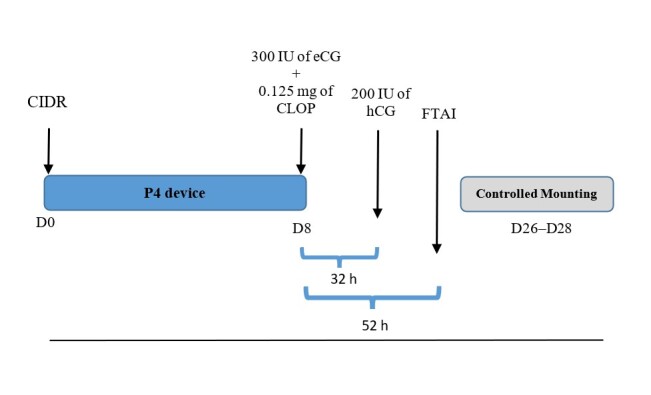
Schematic diagram of the fixed-time artificial insemination (FTAI) protocol and controlled mounting. D0: The day the protocol is initiated, and the progesterone device is introduced (CIDR®, Pfizer, Guarulhos, Brazil). D8: Eighth day of the protocol, removal of the CIDR and application of 300 IU of equine chorionic gonadotropin (eCG, Novormon ®, MSD Saúde Animal, São Paulo, Brazil) and 0.125mg of cloprostenol sodium (Ciosin ®, MSD Saúde Animal, São Paulo, Brazil). 32 h: Ninth day of the protocol, application of 200 IU of human chorionic gonadotrophin (hCG, Chorulon ®, MSD Saúde Animal, São Paulo, Brazil), 32 h after removal of the CIDR. 52 h: Tenth day of the protocol. FTAI is performed 52 h after removal of the CIDR. D26, 27, and 28: Days 16, 17, and 18 after the protocol, estrus is observed and controlled mounting is allowed.

Between days 16 and 18 after the fixed-time artificial insemination protocol, the ewes under the protocol that had returned to estrus (as observed through their acceptance of mating by the ruffian), were serviced by a Santa Inês breeder ram. The last mounting date was duly recorded for subsequent labor induction at 145 days after being serviced or after artificial insemination, as described in Figure 1.

Prior to initiating the reproductive program, the mating ram underwent clinical and andrological evaluations, and his semen was found to be within the evaluation standards recommended by the CBRA (Henry and Neves, 2013). After 30 days of servicing, all ewes underwent an ultrasound to confirm pregnancy and fetal viability, through fetal identification, with observation of heartbeats and fetal movements (Barr, 1988).

Immediately after the evaluations, the pregnant ewes were kept in closed facilities equipped with drinking fountains and troughs with water, hay, and ad libitum mineral salt during the labor-induction period, to facilitate the division between the groups as well as observation of and assistance to the parturient ewes and neonates.

Pregnant ewes were separated into three groups in a completely randomized design. Group G1 (control) received 0.9% NaCl solution via IM injection (n = 19). Group G2 (n = 20) received 8 mg of DEX (Azium®, MSD), and group G3 (n = 19) received 16 mg of DEX (Azium®, MSD). Starting from 24 h after DEX administration, the animals were monitored full-time until day 11 after induction. No human interference between fetal ejection and maternal recognition were allowed, except in cases of dystocia.

After birth and maternal recognition, the neonates’ vitality was assessed based on the modified Apgar score (Silva et al., 2013), postnatal behavior, weight, and temperature, with only one assessment being performed in the first 5 minutes of life.

The following parameters were observed in the Apgar score: heart rate (HR), respiratory rate (RR), mucosa color (MC), general movement (GM), and reflex irritability (RI). The results obtained after adding up the points were interpreted as follows: 7-8 indicated good vitality, 4-6 indicated moderate vitality, and 0-3 indicated low vitality (Silva, 2012). The neonates born in abnormal parturition (dystocia, n = 4) did not undergo neonatal vitality and Apgar evaluation.

For the HR assessment, a score of 0 was assigned when there was no heartbeat, 1 when the frequency was below 100 bpm, and 2 when the frequency was >100 bpm. For RR, a score of 0 was given when the neonates presented with apnea, a score of 1 when RR was under 50 respiratory movements per minute (rpm), and a score of 2 when RR > 50 rpm. MC was assessed by attributing a score of 0, 1, and 2 for cyanotic, pale, and pink mucous membranes, respectively. GM was observed from the moment of birth to the neonate’s contact with the mother: a score of 0 was assigned when there was no movement, 1 when movement was slow, and 2 when movement was vigorous. RI was evaluated through painful stimulus in the interdigital space of the thoracic limb: a score of 0 was given when there was no withdrawal of the limb, a score of 1 for subtle withdrawal, and a score of 2 for quick withdrawal. Table 1 provides the details of the assessment of these parameters.

**Table 1:** Modified Apgar score for sheep.

**VARIABLES**	**SCORE**		
**0**	**1**	**2**
HR	Absence	< 100	>100
RR	Apnea	< 50	>50
MC	Cyanotic	Pale	Pink
GM	Immobile	Not much movement	Vigorous movement
RI	Non-withdrawal of limb	Subtle withdrawal	Rapid withdrawal

HR: heart rate per minute; RR: respiratory rate per minute; MC: mucosa color; GM: general movement; RI: reflex irritability. **Source:** Adapted from (Silva, 2012).

Postnatal behavior was assessed by measuring the intervals, in minutes, between birth and the lamb’s adoption of the sternal decubitus (ISD) position, between birth and the lamb’s adoption of the quadrupedal position (IQP), between birth and the lamb’s pursuit of the mammary gland for the first suckle (IFS). A digital thermometer was further used to measure rectal temperature, a digital scale with a 120-kg capacity was used to weigh the neonate, and the sex was observed.

The SAS v9.1.3 (SAS Institute, Cary, NC, USA) software was used to analyze the variables. Data consistency and descriptive analysis (means and standard deviation) of the characteristics of interest to the study were performed using the MEANS procedure (PROC MEANS). The data were initially tested for normality (Shapiro wilk test). The effect of groups (G1: control, G2: 8 mg DEX and G3: 16 mg DEX), location, sex and type of birth (single or multiple) on neonatal vitality characteristics parameters (temperature, weight, ISD, IQP and IFS) were studied using univariate analyses, for variables that presented an abnormal distribution, the Kruskal - Wallis test was used and for those that presented a normal distribution, ANOVA was used. In order to verify whether the APGAR score is related to indices such as ISD, IQP, IFS, temperature and live weight, the Spearman correlation was performed as these variables were not normally distributed. For all analyses, a significance level of 5% was used.

## Results

Fifty-eight ewes were enrolled in this study, 39 of which underwent labor induction with DEX. The parturitions in the induced groups occurred on average at 147 days of gestation. The parturitions in the non-induced group occurred on average at 150 days of gestation. A total of 79 lambs were born, including 40 females and 38 males; one lamb’s sex was not recorded. Forty lambs were born as multiple births (two or more) and 39 from single births.

Four ewes presented with dystocia: in these cases, the fetal position was in the anterior longitudinal, dorsal position, and flexed attitude of the carpal joint. All cases of dystocia occurred in single births of male lambs. Therefore, 10.25% of the single births and 10.5% of the males were born in dystocia, with two births each from G1 and G2. In these cases, obstetric interventions were necessary to correct the attitude, and forced traction was performed afterwards. All offspring were born alive.

Farm location, type of birth (multiple or single), and sex had no effect on the parameters studied (P > 0.05). The 8- and 16-mg doses of DEX were both efficient at inducing birth in Santa Inês ewes at 145 days of gestation.

The parameters observed in neonates after birth, such as temperature, Apgar score, weight, and postnatal behavior (assuming the ISD position, the standing position, and first suckle), were similar (P>0.05) between groups (Table 2). When verifying the relationship between the APGAR score and the ISD, IQP, IFS, temperature and live weight indices, no significant correlations were found (P>0.05).

**Table 2:** Mean and standard deviation of weight, Apgar score, temperature, and postnatal behavior among groups whose labor was induced with dexamethasone.

	**Doses of dexamethasone**		
	**0 mg (n = 22)**	**8 mg (n = 28)**	**16 mg (n = 25)**
Neonatal weight (kg)	3.5 ± 0.7	3.5 ± 0.8	3.4 ± 0.6
Apgar score	9.8 ± 0.4	9.8 ± 0.5	9.6 ± 0.9
Temperature (°C)	38.7 ± 0.8	39.1 ± 0.7	38.8 ± 0.7
ISD (min)	3.0 ± 3.5	2.7 ± 2.1	2.9 ± 1.8
IQP (min)	19.0 ± 10.8	17.3 ± 10	19.9 ± 12.7
IFS (min)	49.4 ± 30.0	45.3 ± 26.4	57.5 ± 56

ISD: time interval from birth to assuming sternal decubitus position; IQP: time interval from birth to assuming standing position; IFS: time interval from birth to first suckle. Values did not differ by the Kruskal-Wallis test (P > 0.05).

## Discussion

The animals in G1 lambed at an average of 150 days of gestation, which corresponds to the physiological norm for the species. Machado and Simplício (1998) and Silva et al. (1995) respectively found that parturition occurred at an average of 149.80 and 151.66 days of gestation for the Santa Inês breed.

In G3, a 16-mg dose of DEX per animal was chosen based on studies previously conducted by Kastelic et al. (1996) and Ingoldby and Jackson (2001), which established this as the standard dose for inducing labor in sheep (Sir and Bartlewski, 2010). Animals in G2 and G3 gave birth on average at 147 days of gestation, indicating that both doses of DEX were efficient at inducing and concentrating labor.

Labor induction with DEX simulates natural labor. The glucocorticoid mimics the cortisol produced by the fetal adrenal cortex, acting as a trigger to the hormonal cascade that triggers labor (Silver, 1992). Fetal cortisol induces the transformation of placental progesterone (P4) into estrogen (E2), resulting in a reduction in P4 levels and an increase in E2 levels. Estrogen induces the release of prostaglandins, mainly PGF2α, PGE_2_, and PGI_2_, from the uterus. These promote luteolysis, increased placental vascular perfusion, myometrial contractions, and relaxation of the cervix and vaginal canal, triggering parturition (Silver, 1992; Jenkin and Young, 2004).

The fetus releases cortisol naturally from 140 days of gestation. During this period, important processes modulated by cortisol occur, preparing the fetus for post-uterine life (Ingoldby and Jackson, 2001; Feitosa et al., 2017). At 146 days of gestation, fetal cortisol expression peaks, suggesting that even a small dose of DEX would be sufficient to reinforce endogenous cortisol and trigger labor soon after (Tsiligianni et al., 2008), which explains the similar performance between the G2 and G3 groups, even with half the dose.

The weights of the neonates born in the different groups, even in the G2 and G3 groups whose births occurred 2-3 days earlier than that of neonates in G1, did not differ significantly. The weights recorded in the study corroborate reports of the average birthweight of Santa Inês lambs, which is in the range of 3.3-3.6 kg (Castro et al., 2012). This parameter is of paramount importance, since birthweight is correlated with neonatal death (Dwyer and Morgan, 2006). Lower-weight lambs are less vigorous at birth, take longer to suckle colostrum effectively, and are less able to maintain their body temperature than are heavier lambs (Dwyer, 2008). Induction that anticipates labor could negatively influence birthweight.

In this study, lambs born by induced labor presented the same Apgar score classification as non-induced lambs. All the lambs from all groups exhibited good vitality. This finding can be explained by the fact that glucocorticoids such as DEX produce a series of hormonal reactions in the fetus that aid fetal maturation, including maturation of the lungs, liver, kidneys, and intestine (Fowden et al., 1998; Regazzi et al., 2022). In fact, due to these effects, DEX is widely used to aid fetal maturation in situations where the fetus needs to be born prematurely (Whittle et al., 2001).

The lambs’ temperature after birth was within the normal range for the species and did not differ (P > 0.05) among the groups. Thermoregulation is an important factor after birth, and the energy demand to maintain body temperature depends mainly on the heat produced by the oxidation of brown adipose tissue (BAT). Oxidation occurs by controlling triiodothyronine (T3), whose activity increases in the last month of gestation (Bispham et al., 2002; Dauncey, 1990; Forhead et al., 2007).

The neonate’s thermoregulatory capacity enhances with administration of corticosteroids to the mother. Even exogenous administration is an important regulator of fetal maturation, as it increases the production of T3, thereby activating BAT oxidation and consequently raising neonatal temperature (Osathanondh et al., 1978; Bispham et al., 2002; Forhead et al., 2007). Furthermore, it enables the neonate to perform thermogenesis without requiring muscle tremors (Bispham et al., 1999). Thus, all these events explain why the same (P > 0.05) temperature was observed between the lambs in all three groups. After evaluating the influence of sex, number of offspring, and type of birth on the lambs’ neonatal vigor, Fagundes et al. (2019) found temperatures of 38-39 °C to be the normothermic range indicating adequate development of homeostasis mechanisms. These results corroborate the neonatal temperatures reported in this study.

In this study, the neonates’ behavioral chronology after birth was to assume the ISD position first, then the IQP position, and finally suckle at 2.8 ± 2.5, 18.63 ± 11, and 50.49 ± 38 min, respectively. These data were similar to those described by Dwyer (2003) for neonates from eutocic parturitions. The data also corroborated the findings of Barreto et al. (2021), who used the modified Apgar score to assess the influence of maternal and neonatal conditions on lambs’ vitality and survival, and who noted averages in minutes between the ISD position and the first suckle that were similar to those found in this study in neonates with moderate to high Apgar scores.

Regardless to which experimental group they belonged, the neonates presented parameters within the normal range for the species immediately after birth (Dwyer, 2003; Nowak and Poindron, 2006). By pursuing the udder within the first hour of life, they obtained colostrum at the proper time for its absorption, considering that the best use of colostrum occurs within the first 8 hours after birth (Nowak and Poindron, 2006). Thus, the lambs born by labor induction in this study did not exhibit any type of alteration regarding neonatal vitality, based on postnatal behavior, Apgar score, weight, or rectal temperature.

## Conclusions

Neonates born by induced deliveries at 145 days of gestation, using doses of 8 mg or 16 mg of DEX, showed the same vitality as neonates born by non-induced deliveries. Thus, it is concluded that the use of antenatal corticosteroid therapy does not influence the vitality of ovine neonates, implying that these DEX doses can be used safely to induce labor in ewes.
